# Production of 4-Ethyl Malate through Position-Specific Hydrolysis of *Photobacterium lipolyticum* M37 Lipase

**DOI:** 10.4014/jmb.2112.12055

**Published:** 2022-03-17

**Authors:** Chae Ryeong Lim, Ha young Lee, Ki-Nam Uhm, Hyung Kwoun Kim

**Affiliations:** 1 Division of Biotechnology, The Catholic University of Korea, Bucheon 14662, Republic of Korea; 2 C1Chem Co, Ltd., 405 Sogang Business Center, Seoul 04107, Republic of Korea

**Keywords:** 4-ethyl malate, ArF photoresist, M37 lipase, position-specificity

## Abstract

Microbial lipases are used widely in the synthesis of various compounds due to their substrate specificity and position specificity. 4-Ethyl malate (4-EM) made from diethyl malate (DEM) is an important starting material used to make argon fluoride (ArF) photoresist. We tested several microbial lipases and found that *Photobacterium lipolyticum* M37 lipase position-specifically hydrolyzed DEM to produce 4-EM. We purified the reaction product through silica gel chromatography and confirmed that it was 4-EM through nuclear magnetic resonance analysis. To mass-produce 4-EM, DEM hydrolysis reaction was performed using an enzyme reactor system that could automatically control the temperature and pH. Effects of temperature and pH on the reaction process were investigated. As a result, 50°C and pH 4.0 were confirmed as optimal reaction conditions, meaning that M37 was specifically an acid lipase. When the substrate concentration was increased to 6% corresponding to 0.32 M, the reaction yield reached almost 100%. When the substrate concentration was further increased to 12%, the reaction yield was 81%. This enzyme reactor system and position-specific M37 lipase can be used to mass-produce 4-EM, which is required to synthesize ArF photoresist.

## Introduction

Lipases (E.C. 3.1.1.3) are enzymes that can hydrolyze fats to produce glycerol and fatty acids [1, 2]. Lipase is an industrially useful enzyme catalyst because it can hydrolyze triglycerides with various fatty acid chains. It can also catalyze various ester syntheses or transesterification in non-aqueous systems [3, 4]. So far, many lipases have been isolated from various animals, plants, and microorganisms. Their reaction characteristics and protein structures have also been revealed. In particular, it has been found that microbial lipases have various reaction properties depending on microbial sources [5, 6]. That is, they have substrate specificity according to fatty acid length and unsaturation degree. They also have position specificity and stereo-specificity.

Meanwhile, all lipases whose structures have been identified so far have a typical α/β hydrolase fold in common and a catalytic triad composed of Ser, His, and Asp at the active site [7-9]. Substrate/position specificities of each microbial lipase are determined by amino acids surrounding the active site pocket and ‘lid’ structure [10]. The reaction mechanism of lipase has been explained at the molecular level through studies of the X-ray crystal structure and the docking model of the lipase-ligand complex [11].

Lipase is widely used as an enzyme catalyst in food, agriculture, chemical, pharmaceutical, cosmetic, environment, and energy industries. For example, it is used in the synthesis of specific fats, flavoring ingredients, pharmaceuticals, antioxidants, antimicrobial agents, and biodiesel [12-15]. In addition, lipase itself is a component of detergents and digestive drugs [16-18]. In order to industrially utilize lipase, it is necessary to select and use a suitable lipase according to the type of substrate and reaction conditions.

In general, when an ester compound is synthesized using a chemical catalyst, the reaction rate is fast and the catalyst price is low. However, there are problems in that the reaction must be performed at a high temperature and unwanted by-products are generated. In addition, a large amount of environmental pollutants must be used [19, 20]. On the other hand, in the case of using a lipase catalyst, the amount of by-products can be reduced by performing the reaction at a mild temperature and selectively synthesizing only desired materials using substrate-, position-, and stereo-specific lipases. Therefore, an environment-friendly enzymatic process is increasingly used in industry [21, 22].

**Fig. 1:**
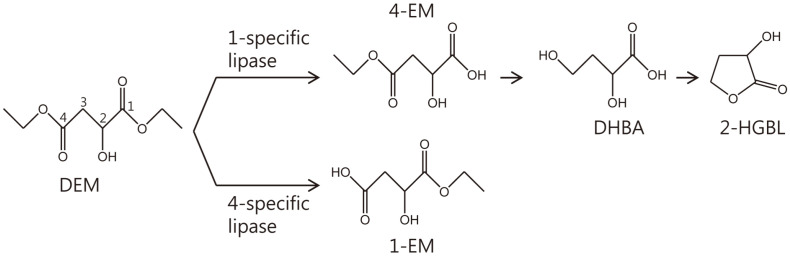
Scheme of 2-hydroxy-γ-butyrolactone (2-HGBL) production. 4-Ethyl malate (4-EM) is produced from diethyl malate (DEM) using position1-specific lipase. 4-EM is used for the production of 2-HGBL, an intermediate of ArF photoresist. DHBA: 2,4-dihydroxy butyric acid.

Photoresist is a polymer material whose resistance to chemicals is changed by exposure to light. It is an important material used for manufacturing semiconductors. A commonly used polymer is argon fluoride (ArF) photoresist. To produce ArF, 2-hydroxy-γ-butyrolactone (2-HGBL) as its precursor is required [23, 24]. If we find and use a lipase that can specifically hydrolyze only the 1-position of the 1,4-diethyl malate (DEM) molecule, we can produce high-purity 4-ethyl malate (4-EM). 4-EM can be converted to 2-HGBL via dihydroxybutyric acid (DHBA) through chemical and enzymatic processes (Fig. 1).

In this experiment, it was confirmed that *Photobacterium lipolyticum* M37 lipase could hydrolyze substrate DEM to produce 4-EM position-specifically. An enzyme reactor system for mass production of 4-EM was then developed and an experiment was performed to optimize the enzyme reaction.

## Materials and Methods

### Materials

Four genes (*Photobacterium lipolyticum* M37 lipase [25-28], *Proteus vulgaris* K80 lipase [29], *Bacillus stearothermophilus* L1 lipase [30], *Staphylococcus haemolyticus* L62 lipase [31]) isolated previously by our laboratory were used in this study. *Candida antarctica* lipase B (CalB) enzyme was purchased from Novozyme. Alcalase (*Bacillus licheniformis* protease), diethyl malate, p-nitrophenyl caprylate, silica gel (Davisil Grade 635, pore size 60Å, 60-100 mesh), and 1-butanol were purchased from Sigma-Aldrich. Acetic acid was purchased from Kanto. Potassium permanganate and formic acid were purchased from Daejung. Sodium hydroxide and potassium carbonate were purchased from Jusei. Acetonitrile was purchased from Duksan.

### Expression of Lipases and Preparation of Cell-Free Extracts

Four recombinant pET22 plasmids containing M37, K80, L1, and L62 lipase genes were electroporated into *Escherichia coli* BL21 (DE3) cells and cultured on LB-ampicillin (100 μg/ml) agar plates. Transformed *E. coli* was inoculated into LB-ampicillin broth and cultured at 37°C with shaking (200 rpm). When OD_600nm_ of the culture was 0.5, IPTG was added at a concentration of 1 mM and cultured at 20°C for 20 h. Cells were recovered from the culture through centrifugation (4,000 ×*g*, 10 min) and suspended in 50 mM potassium phosphate buffer (pH 7.0). After cells were disrupted by ultrasonication, the supernatant obtained by centrifugation (1,000 ×*g*, 10 min) was used as a lipase enzyme solution.

### Lipase Activity Assay

Enzyme activity measurement using a synthetic substrate was performed as follows. p-Nitrophenyl caprylate (PNPC) (dissolved in acetonitrile at a concentration of 10 mM), ethanol, and 50 mM potassium phosphate (pH 7.0) buffer were mixed at a ratio of 10:40:950 and 1 ml of this mixture was used as a substrate. A small amount of enzyme solution was added to this mixture, reacted at 37°C for 3 min. The amount of p-nitrophenol (PNP) produced was determined by measuring the absorbance at 405 nm and quantified. Enzyme activity of 1 unit was defined as the amount of enzyme that produced 1 μmol of PNP in 1 min.

### Lipase Reaction and Thin Layer Chromatography Analysis

DEM hydrolysis was performed using six enzymes (M37, K80, L1, L62, CalB, and Alcalase) as follows. Potassium phosphate (50 mM, pH 7.0) buffer 890 μl, DEM 10 μl, and lipase 100 μl were placed in a tube (2 ml). The concentration of DEM (1%, v/v) corresponded to 53 mM. The tube was placed in an incubator/shaker. The reaction was performed while rotating the tube at 200 rpm for 2 h at 30°C (L1 was reacted at 50°C).

After the enzymatic reaction, TLC analysis was performed to qualitatively analyze amounts of substrate and the reaction product. A 1.5 μl volume of the reaction solution was dropped on a silica gel plate (60 F_254_) and dried. As a TLC developing solvent, a mixture of 1-butanol, acetic acid, and water at 40:10:10 was used. After TLC development, the dried silica gel plate was immersed in a color reagent (1 g of KMnO_4_, 6.67 g of K_2_CO_3_, and 1.6 ml of 5% NaOH in 100 ml of distilled water) and dried. After color development, the substrate and reaction product spots on the silica gel plate were observed.

### High Performance Liquid Chromatography Analysis

To quantitatively measure the lipase reaction product, HPLC analysis was performed. The reaction solution (200 μl) was sampled for each predetermined time and filtered using a nylon syringe filter (0.2 μm). As the analytical column, a Cogent Bidentate C_18_ column (4.6 mm × 250 mm, 5 μm particle size; microSolv Technology Corp., USA) was used. After injecting 10 μl of the filtrate into the column, the reaction product and substrate that passed through the column were analyzed through a UV detector (220 nm). As HPLC solvents, solution A (100%water, 0.1% formic acid) and solution B (70% acetonitrile, 30% water, 0.1% formic acid) were used. After injection, solution A and solution B were added into the column according to a predetermined program: 0–2 min (10% B, isocratic), 2–15 min (10-100%B, gradient), 15–18 min (100-10%B, gradient), and 18–25 min (10%B, isocratic). From the HPLC chromatogram, peak areas of the substrate before reaction (A_DEM, 0h_) and after reaction (A_DEM, t_) were obtained. The conversion yield of the enzyme reaction was calculated according to the following equation:

$$\text{Converion yield (\%) = }\frac{{\text{A}}_{\text{DEM,Oh}}{\text{-A}}_{\text{DEM,t}}}{{\text{A}}_{\text{DEM,Oh}}}\text{×100}$$

### Purification of Reaction Product and Nuclear Magnetic Resonance Analysis

Silica gel chromatography was performed to isolate 4-EM. A gel slurry was prepared by mixing silica gel powder with acetonitrile. The silica gel slurry was poured into a column (15 mm × 300 mm) and the column was washed with acetonitrile. A reaction solution (1 ml) obtained by performing DEM hydrolysis for 2 h using M37 enzyme was then added to the column. While acetonitrile was continuously added to the column, 1 ml each of the solution eluted from the column was obtained in 25 tubes. The tube containing 4-EM was found by adding each fraction to a TLC plate followed by a color reaction. In addition, the tube containing 4-EM was quantitatively measured through HPLC analysis. After collecting all fractions containing 4-EM, the solvent was evaporated through rotary evaporation concentration.

^1^H-NMR analysis was performed to confirm the chemical structure of the compound purified through silica gel chromatography. After adding 600 μl of CDCl_3_ to samples, ^1^H-NMR (300 MHz Nuclear Magnetic Resonance) analysis was performed. The chemical structure of the purified compound was confirmed by comparing NMR peaks of DEM, 1-EM, and 4-EM obtained with a ChemDraw program using actual NMR peaks obtained.

### Effects of Temperature and pH on DEM Hydrolysis Yield

To investigate the effect of temperature on DEM hydrolysis, an experiment was performed in two ways. First, potassium phosphate (50 mM, pH 6.0) buffer (890 μl), DEM (10 μl), and M37 solution (100 μl) were placed in a reaction tube (2 ml). The reaction tube was placed in water bath. The reaction was performed for 30 min at various temperatures (10°C–70°C). HPLC analysis was then performed. The effect of temperature in a 20 ml reaction system was investigated using a reactor. Potassium phosphate (100 mM, pH 6.0) buffer solution (20 ml), DEM (400 μl), and M37 (500 μl) were put into a reactor and reaction was performed at 30°C, 40°C, or 50°C. HPLC analysis was performed by sampling the reaction solution for predetermined time while the reaction was carried out for 5 h.

Buffer solutions with various pH values were used to investigate the effect of pH on DEM hydrolysis, including sodium citrate (100 mM, pH 2.5–6.0) and potassium phosphate (100 mM, pH 6.0–8.0). Buffer (20 ml), DEM (400 μl), and M37 (500 μl) were added into the enzyme reactor and the temperature was adjusted to 50°C. HPLC analysis was performed by sampling the reaction solution at predetermined time while the reaction was performed for 4 h.

### Effect of Substrate Concentration on DEM Hydrolysis Yield

In order to investigate the effect of substrate concentration on DEM hydrolysis, the reaction was performed as follows. Sodium citrate (100 mM, pH 4.0) buffer (20 ml) and M37 (500 μl) were added to the reactor. DEM at 2% to 12% was then added to the reactor. The reaction was performed at 50°C. The reaction solution was sampled at predetermined time while the reaction was performed for 4 h. HPLC analysis was performed and the yield of the reaction was calculated.

## Results and Discussion

### Lipase Screening for Position-Specific Hydrolysis of DEM

In DEM hydrolysis, 1-EM or 4-EM is produced depending on the position specificity of the lipase (Fig. 1). Since 2-hydroxy-γ-butyrolactone (2-HGBL), a raw material of photoresist, is synthesized using 4-EM, it is necessary to screen and use a position 1-specific lipase.

The enzyme solution was prepared for four lipases of M37, K80, L1, and L62 available from our laboratory. Each of these four lipases was produced from recombinant *E. coli* BL21 (DE3). Cell extracts were prepared. The lipase activity of each cell extract was measured through PNPC assay. As a result, it was found that the lipase activity was 21 U/ml for M37, 130 U/ml for K80, 657 U/ml for L1, and 27 U/ml for L62, with L1 showing the highest lipase activity and M37 showing the lowest activity.

**Fig. 2:**
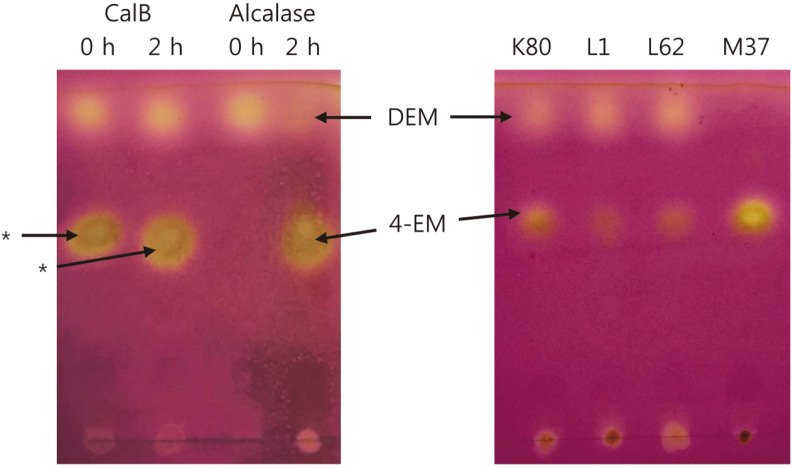
TLC of enzyme reaction products. CalB, Alcalase, K80, L1, L62, and M37 were used as catalysts for the hydrolysis of DEM. After a 2 h-reaction, each reaction mixture was applied to a TLC plate, developed, and stained. CalB, *Candida antarctica* lipase B; K80, *Proteus vulgaris* lipase; L1, *Bacillus stearothermophilus* lipase; L62, *Staphylococcus haemolyticus* lipase; M37, *Photobacterium lipolyticum*; *, stabilizer of CalB enzyme.

In order to search for lipases having high reactivity with DEM, reactivities of the above four laboratory lipases and two commercial enzymes (CalB, Alcalase) were measured. DEM 1% and potassium phosphate (50 mM, pH 7.0) buffer were used. Then 100 μl of M37, K80, L1, or L62 lipase, or 20 μl of CalB or Alcalase was added to make a 1 ml reaction solution. The reaction was carried out for 2 h. When the reaction solution was analyzed by TLC, it was confirmed that the DEM spot was greatly reduced and the reaction product spot was generated in the reaction with K80, M37, or Alcalase. When *E. coli* BL21 (DE3) cell-free extract containing L1 lipase was used, DEM degradation hardly proceeded. This is evidence that *E. coli* BL21 (DE3) itself did not have DEM hydrolytic activity. In the case of CalB, it was confirmed that 0 h and 2 h spots were the same on TLC, confirming that the lower spot was an enzyme stabilizer (Fig. 2). In the case of M37, the biggest product spot was produced by hydrolyzing all DEM.

Based on the results of PNPC measurement and TLC analysis, it was found that M37 showed the lowest PNPC hydrolysis activity but the highest DEM hydrolysis activity among the four lab lipases. In general, lipases have different hydrolytic activities depending on the substrate. This is because the kinetic parameters (*k*_cat_ and *K*_M_) of each lipase towards substrates are very different. From the above results, it could be concluded that M37 has a lower *k*_cat_/*K*_M_ for PNPC, but a higher *k*_cat_/*K*_M_ for DEM than the other three enzymes. In addition, since the DEM hydrolysis yield of M37 was the highest, even higher than that of CalB or Alcalase based on TLC analysis, the next experiment was performed using M37.

### HPLC Analysis of Reaction Solution

**Fig. 3:**
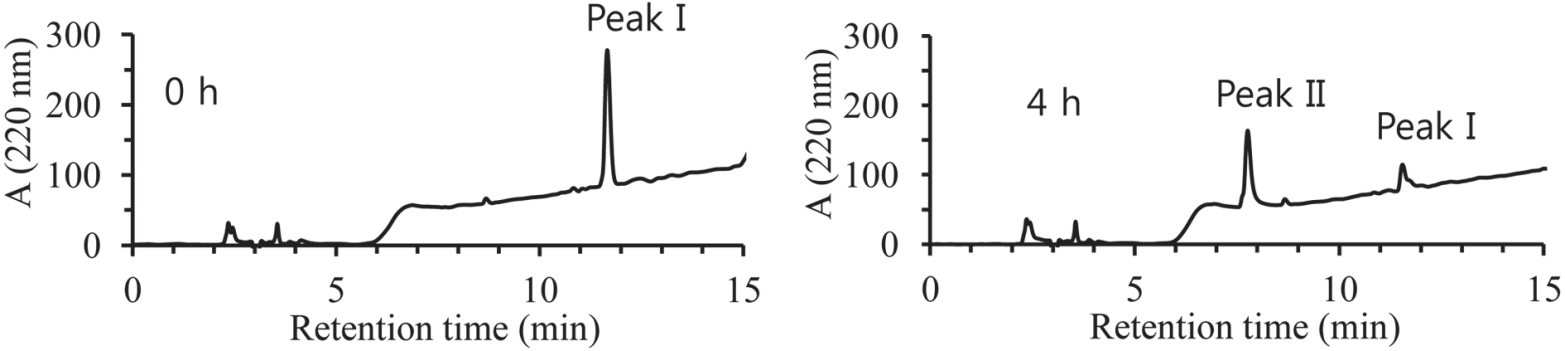
HPLC of enzyme reaction products. Reaction mixture were analyzed using HPLC. Peak I (DEM) and peak II (4-EM) were eluted at 11.6 min and 7.6 min, respectively.

After performing DEM hydrolysis using M37, a quantitative analysis of the reaction solution was performed using HPLC. After sampling reaction solutions at 0 h and 4 h, the substrate and reaction product were analyzed using HPLC. For the 0 h sample, a peak at 11.6 min (peak I) was observed. For the 4 h sample, peak I and a new peak at 7.6 min (peak II) were observed (Fig. 3). Accordingly, it could be seen that peak I was DEM and peak II was a reaction product. On the other hand, a chemically synthesized 1-EM and 4-EM mixture showed two peaks at 7.5 min and 7.6 min (data not shown). Among them, it was later confirmed through NMR analysis that the peak at 7.6 min was 4-EM. Accordingly, it was found that M37 produced only 4-EM by selectively hydrolyzing the 1-position ester bond of DEM.

### Purification of Reaction Product and NMR Analysis

To analyze the chemical structure of the compound showing peak II, the compound was purified from the reaction solution using a silica gel chromatography. After performing the reaction for 4 h, 1 ml of the reaction solution was added to the top of the silica gel column followed by column elution using acetonitrile. Fractions (1 ml each) coming out of the column were collected into 25 tubes and were spotted onto TLC plates. Five fractions (No. 11 to No. 15) showed color reactions. As a result of performing HPLC analysis for these five fractions, peak I was not observed. Only peak II corresponding to 4-EM was shown.

**Fig. 4:**
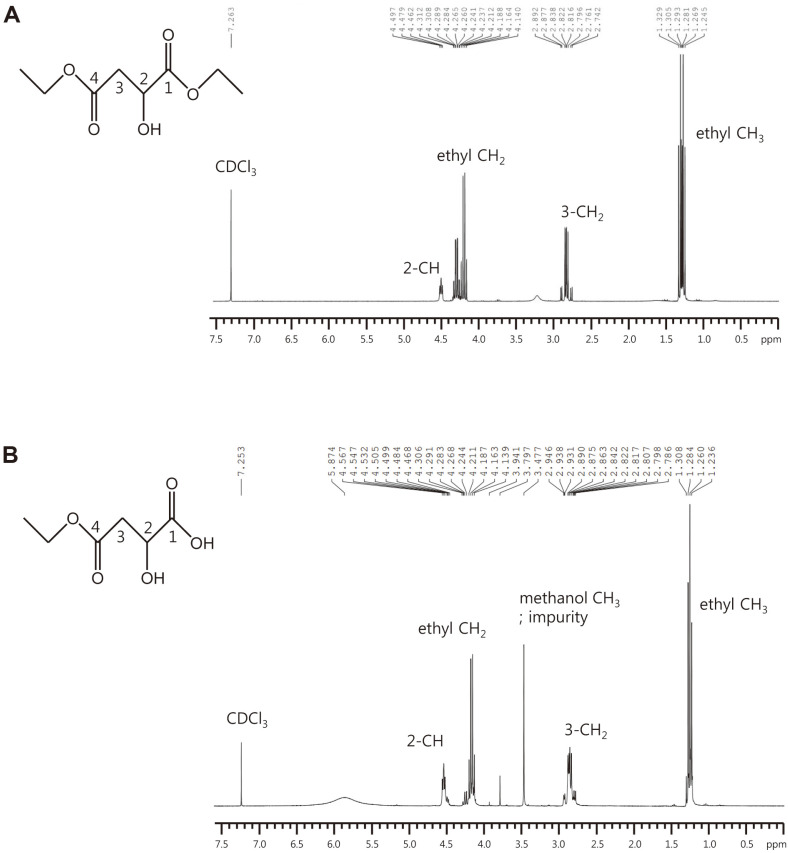
^1^H-NMR (CDCl_3_) spectrum of 4-EM purified by silica gel column chromatography. NMR spectra were measured with an Avance III 300 MHz (Bruker BioSciences Corp., USA). **A**, ^1^H-NMR of DEM; **B**, ^1^H-NMR of reaction product.

After collecting all solutions in five fractions, acetonitrile was evaporated through rotary evaporation concentration and ^1^H-NMR analysis was performed. First, we analyzed the NMR results of DEM (Fig. 4A). Results are shown below: δ, (1.245–1.329) (m, 6H, ethyl 2CH_3_), (2.742–2.892) (m, 2H, 3-CH_2_), (4.140–4.312) (m, 4H, ethyl 2CH_2_), (4.462–4.497) (m, 1H, 2-CH). NMR results for peak II are as follows (Fig. 4B): δ, (1.227–1.384)(m, 3H, ethyl CH_3_), (2.773–2.925) (m, 2H, 3-CH_2_), 3.461 (s, 3H, methyl CH_3_, impurity), (4.127–4.199) (m , 2H, ethyl CH_2_), (4.478–4.555) (m, 1H, 2-CH). Meanwhile, as a result of comparing the predicted NMR of DEM, 1-EM, and 4-EM through the ChemDraw Ultra program (Cambridgesoft), ppm values of all hydrogens were similar, although there was one difference in the ppm value of the hydrogen atoms bound to position 2 carbon (2-CH). That is, 4-EM, DEM, and 1-EM were predicted to have 4.53, 4.48, and 4.18 ppm peaks, respectively. Our experimental results confirmed that peak II was 4-EM because it had a hydrogen ppm value of 4.4784.555, whereas DEM had the ppm value of 4.4624.497.

### M37 Lipase Reaction Using an Enzyme Reactor

**Fig. 5:**
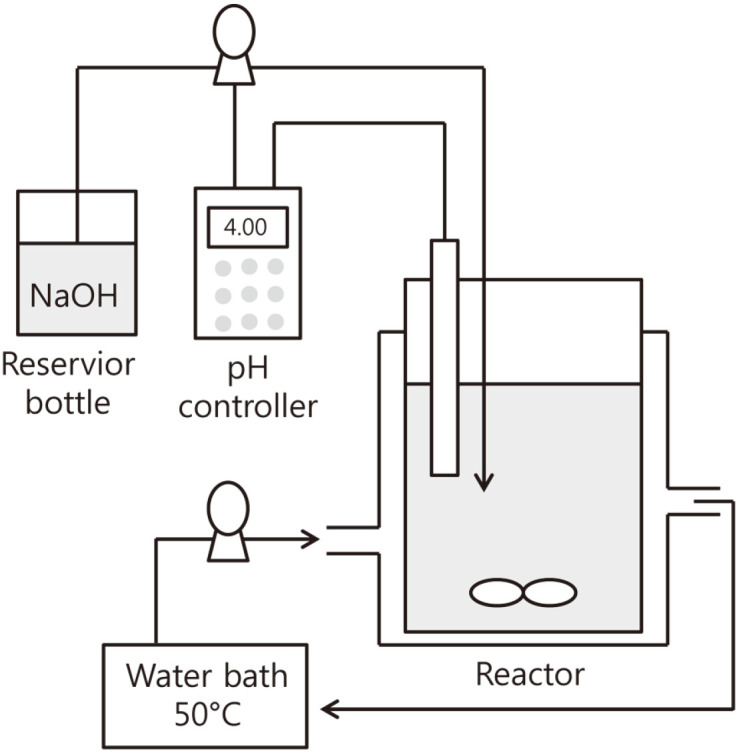
pH-controlled enzyme reaction system for the production of 4-EM. Temperature of reaction bottle was controlled through outer jacket connected to water bath. pH of reaction bottle was controlled using pH controller connected to a NaOH reservoir.

Based on information obtained through tube reactions (1 ml), an enzyme reaction with a volume of 20 ml was designed using an reactor system (Fig. 5). The reason why the reactor system was performed was as follows. First, large amounts of HGBL are required to produce a photoresist. Therefore, we attempted to synthesize 4-EM, a precursor of HGBL, in large quantities through a reactor system. Second, we attempted to confirm whether the scale-up of the enzyme reaction was possible. That is, we wanted to confirm whether M37 could stably produce 4-EM even in a reactor system. Third, we wanted to check whether the optimal pH and temperature of the M37 confirmed in the tube reaction could be applied in a scale-up process.

The enzyme reactor system for mass production of 4-EM was as follows (Fig. 5). First, a water bath, a pump, and a water jacket were connected to keep the temperature of the reactor constant. In addition, the pH of the reactor was constantly adjusted using a pH controller and a peristaltic pump. For this purpose, 4% NaOH was automatically added. A stirring device was used to uniformly mix the reaction solution.

As a result of carrying out the reaction by putting DEM and M37 in the reactor, it was confirmed that the pH was continuously decreased and the NaOH solution was automatically supplied accordingly. After a certain period of time, it was confirmed that most DEMs were converted to 4-EM when the pH did not drop anymore.

### Effects of Reaction Temperature and pH on DEM Hydrolysis Yield

**Fig. 6:**
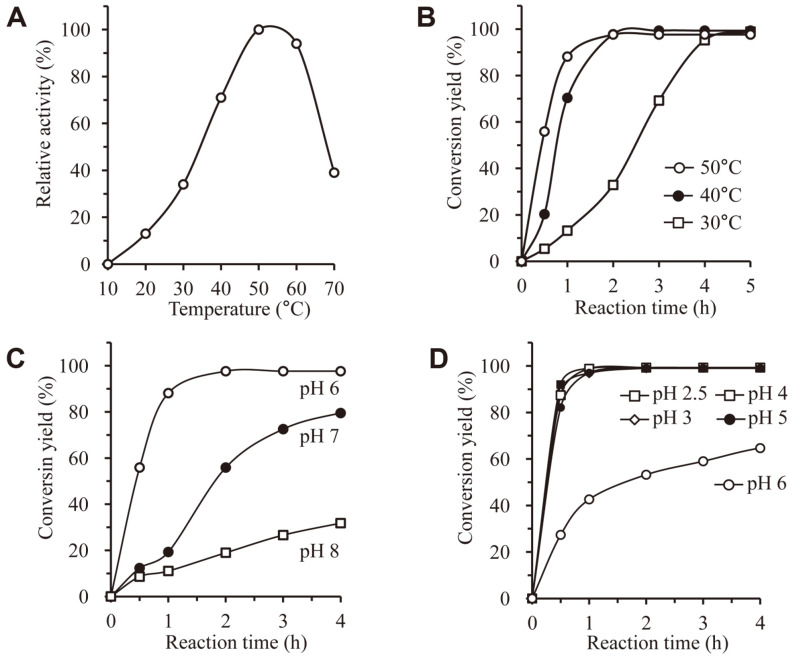
Effects of temperature and pH on the production of 4-EM. **A**. Enzyme reaction was performed in phosphate buffer (pH 6.0) at different temperatures (10–70°C) for 30 min and the amount of 4-EM were analyzed. **B**. Conversion yield were measured with time course at 30–50°C. **C**. Conversion yield were measured with time course at 50°C in phosphate buffer (pH 6–8). **D**. Conversion yield were measured with time course at 50°C in citrate buffer (pH 3–6).

To confirm the optimal temperature of the DEM hydrolysis using M37, the initial reaction rate of the DEM hydrolysis was measured in a tube reaction. After the reaction was performed for 30 min at various temperatures from 10°C to 70°C, HPLC analysis was performed. 4-EM was not detected at 10°C. The amount of 4-EM increased as the temperature increased. At 50°C, most DEMs were converted to 4-EM. The amount of 4-EM decreased as the temperature was increased higher (Fig. 6A).

In addition, the reaction yield according to the reaction time course was measured in the reactor system. We performed reactions at 30°C, 40°C, and 50°C. The initial reaction rate was also high at 60°C in tube reaction. However, we did not conduct the reactor reaction at 60°C because denaturation of the enzyme would proceed when the reaction was carried out for a long time at a high temperature. In addition, the composition of the reaction solution would change due to the evaporation of the buffer.

In such a reactor system, the reaction yield for 5 h was 99% at 30°C, 99% at 40°C, and 98% at 50°C. These yields were very high. However, the yield for 1 h was 13% at 30°C, 70% at 40°C, and 88% at 50°C. Therefore, it was confirmed that the reaction proceeded the fastest at 50°C among the three temperatures used (Fig. 6B). Accordingly, the optimum reaction temperature for M37 was confirmed to be 50°C.

In order to confirm the optimal pH of the DEM hydrolysis using M37, at first, the initial reaction rate of DEM hydrolysis was measured in a tube reaction. After performing the reaction for 30 min using sodium citrate (50 mM, pH 2.5–6.0), potassium phosphate (50 mM, pH 6.0–8.0), or Tris-HCl (50 mM, pH 8.0–9.0), HPLC analysis was performed. As a result of the analysis, a high reaction yield was shown at pH of 3.0 to 6.0 (data not shown).

In addition, the reaction yield according to the reaction time was measured while performing the reaction for each pH in the reactor system. We carried out the reaction from pH 2.5 to pH 8.0 for 4 h using a pH buffer solution at 100 mM. As a result of the analysis, in the case of potassium phosphate (100 mM, pH 6.0–8.0), the reaction yield was 98% at pH 6, 79% at pH 7, and 32% at pH 8 (Fig. 6C). On the other hand, in the case of sodium citrate (100 mM, pH 2.5–6.0), a low reaction yield of 59% was obtained at pH 6 whereas a high reaction yield of 99% was obtained at pH of 2.5 to 5.0 (Fig. 6D). These results showed that the optimal pH of M37 was acidic.

Most microbial lipases studied so far have high activity and stability at neutral pH. Only a few fungal lipases have high activities at acidic pH. For example, *Aspergillus niger* [32], *Rhizophus* sp. [33], and *Candida rugosa* [34] can produce extracellular lipases that are active at acidic pH. Interestingly, although M37 was a typical bacterial lipase, it had a high DEM hydrolytic activity at acidic pH.

Acid lipase provides several advantages to the reaction process in the food and chemical industries. When the reaction process is carried out for a long time, it is easy to be contaminated with microorganisms. However, if the reaction proceeds under acidic conditions, contamination can be prevented. In addition, it is possible to prevent unwanted side reactions by contaminating enzymes. Of course, acidic lipase can be used only in the conversion reaction of a substrate having high solubility and stability in an acidic condition.

In this research, although the reaction yield was slightly higher at pH 3.0, we decided to perform the reaction at pH 4.0 in order to minimize enzyme denaturation during the long reaction process.

### DEM Hydrolysis Yield with Increasing Substrate Concentration

**Fig. 7:**
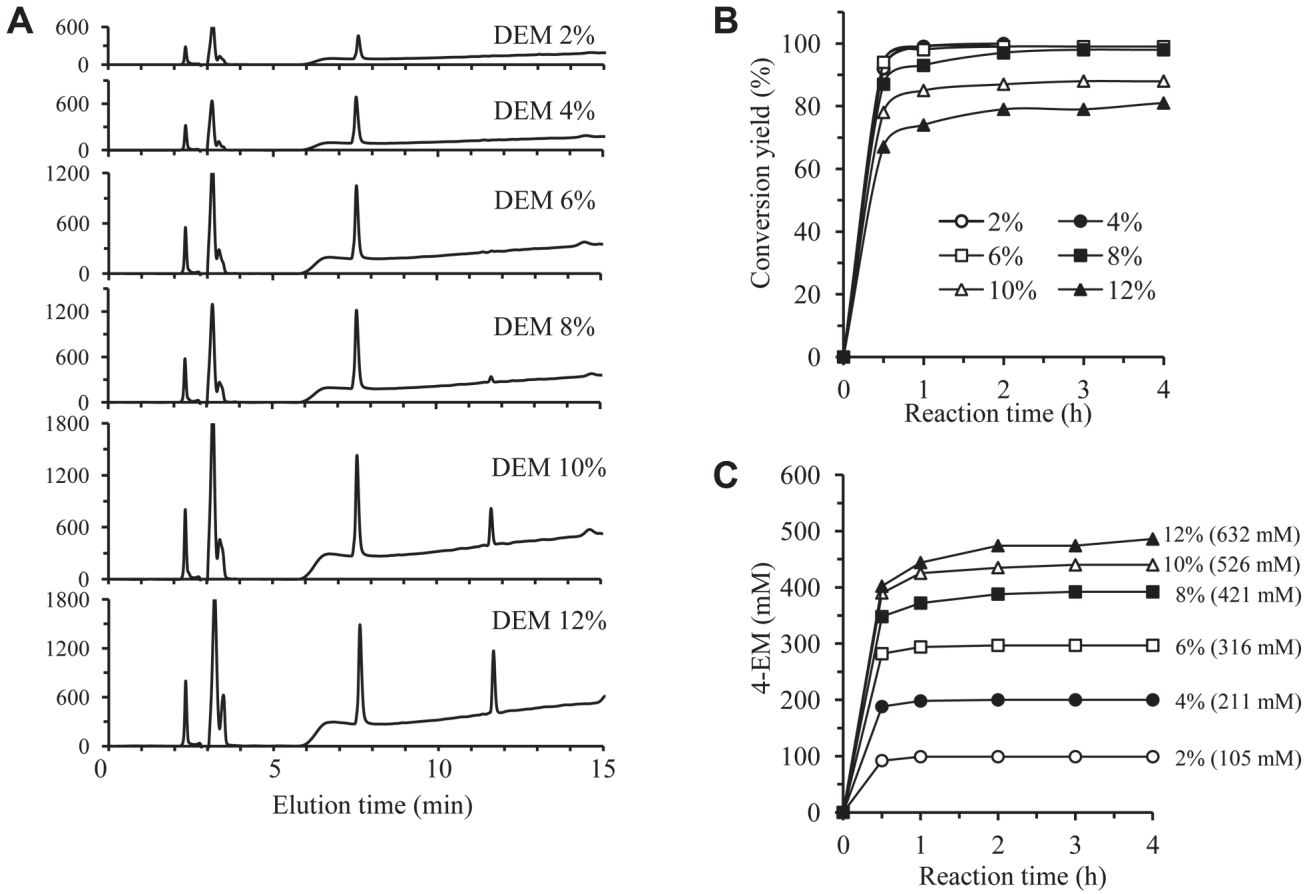
Production of 4-EM using reactor system. Enzyme reaction was performed in 20 ml citrate buffer (pH 4.0) at 50°C using increasing amount of DEM. **A**. HPLC chromatogram of reaction mixture after 2 h-enzyme reaction. **B**. Conversion yields were calculated with time course. **C**. 4-EM concentration was calculated with time course.

In order to investigate the effect of substrate concentration on DEM hydrolysis of M37, the reaction was performed as follows. M37 (500 μl) was added to sodium citrate buffer (100 mM, 20 ml) under optimal reaction conditions (pH 4.0, 50°C). DEM concentration was increased from 2% to 12% in 2% increments. In order to measure the decrease in DEM and the increase in 4-EM by the enzyme reaction, the reaction solution was sampled at different time points (Fig. 7A). In the case of DEM 2-6% reaction, after 1 h of reaction, DEM was almost all decomposed and 4-EM increased in proportion to the DEM concentration. DEM 6% corresponded to a concentration of 0.32 M. Therefore, it was confirmed that 0.32 M of 4-EM could be produced with a high purity using a 20 ml reactor system. In the case of DEM 8%, 10%, and 12% reactions, it was confirmed that conversion yields after 4 h of reactions were 98%, 88%, and 81%, respectively (Fig. 7B).

Through this experiment, we revealed for the first time that M37 from *P. lipolyticum* could selectively produce 4-EM, a raw material for photoresist, by position-specifically degrading DEM. The optimum temperature and pH conditions for the M37 reaction were found to be 50°C and pH 4.0, respectively, using a reactor system. Through this process, it was revealed that M37 was a uniquely acidic lipase. It stably carried out DEM hydrolysis at acidic pH for a long time. In addition, a reactor system for mass production of 4-EM was established by increasing DEM concentration under optimal reaction conditions.
